# Quality evaluation of pregnancy-related mobile applications in South Korea: a descriptive study

**DOI:** 10.4069/kjwhn.2023.06.20

**Published:** 2023-09-26

**Authors:** Hyunjin Cho, Feiyan Yi, Sukhee Ahn

**Affiliations:** College of Nursing, Chungnam National University, Daejeon, Korea

**Keywords:** Mobile applications, Pregnancy, Program evaluation, Quality improvement

## Abstract

**Purpose:**

This study aimed to describe the characteristics of mobile applications (apps) related to pregnancy in South Korea and evaluate their quality.

**Methods:**

We conducted a systematic search for pregnancy-related apps available in Korea in two app stores as of April 29, 2022. The quality of apps was assessed using the Korean translation of the Mobile Application Rating Scale for objective quality with four subdomains (engagement, function, aesthetics, and information) and four items for subjective quality.

**Results:**

In total, 163 apps were selected and reviewed. Both the objective and subjective quality of the apps were found to be desirable, with scores exceeding 3 out of 5 (range, 34–82). All subdomain scores in the objective quality assessment were also desirable. Among the four objective quality subdomains, aesthetics received the highest scores, followed by information, function, and engagement. In terms of subjective quality, the scores for a comprehensive overall evaluation, continuous use, and recommendation exceeded 3 out of 5, with the exception of payment. Only a small number of apps (n=4, 2.9%) were backed by a reliable authority, such as an expert review. Significant differences were observed in the objective quality of apps across different content categories (F=3.86, *p*=.003).

**Conclusion:**

Most pregnancy-related apps had desirable levels of objective and subjective quality. However, app content experts seldom provide reviews. It is crucial for nurses to recommend apps to expectant mothers that offer dependable content, regularly updated with the latest information.

## Introduction

The growing demand for health information has been accompanied by an increasing trend of exploring health-related information using internet searches and mobile applications (apps) [1]. People in their 20s and 30s, who are typically familiar with electronic devices, frequently download and use apps related to health or exercise. Interestingly, men have been found to use exercise apps more frequently than women [2]. With the widespread use of smartphones, access to apps is increasing across all age groups. Notably, 60% of active seniors in their 50s are proactive smartphone users [3]. Apps are now more popular for obtaining health-related information than traditional visits to medical institutions [4]. In Italy, women were found to be more engaged in e-health than men, and younger people demonstrated higher access to and usage of e-health apps [5]. A study revealed that mobile app-based health promotion programs provided individual feedback on health status and monitored health/behavioral changes using apps that focused on diet, physical activity, and a healthy lifestyle. The study also found that app users exhibited better health outcomes than nonusers [6].

Women experiencing pregnancy and childbirth are increasingly moving away from traditional sources of information such as physicians, nurses, family, and friends. Instead, they are turning to e-health platforms like the internet and mobile apps for advice on physical activity and pregnancy [7]. Many pregnant women express a desire for healthcare providers to recommend reliable internet sites for obtaining pregnancy-related information [8]. A prior study revealed that 96% of American women aged between 18 and 49 use smartphones. These devices provide easy access to apps at any time and place and have been shown to positively influence physical activity behaviors, demonstrating the benefits of smartphone apps [9]. For American women navigating pregnancy, childbirth, and the postpartum period, apps have become a widely used resource and communication channel. They offer information on managing health during pregnancy, caring for infants and children, and parenting [10]. Most pregnancy and childbirth-related apps fall under the categories of health/fitness, medical care, or education. Their functions typically include information provision, education, tips or advice, pregnancy tracking and monitoring, meditation, and goal achievement [11]. Pregnancy-related mobile health apps can motivate individuals to adopt lifestyle changes that promote optimal health during pregnancy. They provide necessary information and support decision-making [7]. Furthermore, these apps serve as a conduit for accurate information and behaviors related to caring for their children. This includes raising awareness among pregnant women about reduced fetal movement, weight monitoring, and breastfeeding [12]. The most common reasons for seeking pregnancy-related information on the internet include the need to enhance knowledge about pregnancy, insufficient information from healthcare providers [13], anonymity, rapid search capabilities, and the convenience of access at any time and place [14].

The proliferation of health information through mass media and the internet has led to an increase in concerns about the credibility and accuracy of the information provided. Pregnant women who rely on internet-sourced information often perceive the quality of the information they find as good or very good. The majority find it useful, with over 50% reporting a significant influence on their decision-making processes [15]. However, there is a high risk of exposure to unverified information due to a lack of proper scrutiny regarding the quality and reliability of the information. This can lead to unnecessary worry or misinformation [7], especially when the information is not discussed with healthcare providers [16]. It is important to exercise caution as inaccurate information disseminated through apps can potentially harm pregnant women and their fetuses [17]. The responsibility for the accuracy of the content in pregnancy and childbirth apps lies with the developers. Currently, there are no regulations addressing inappropriate information or uncertain evidence, and the guidelines for app development are significantly lacking in medical-related industrial regulatory criteria [18]. Recent studies assessing apps have highlighted that many health apps are rarely reviewed or approved by healthcare providers, nor do they have peer review systems in place to ensure the content and quality of information [18,19]. Moreover, while the benefits of apps that support decision-making during pregnancy are emphasized, it has been noted that there is a lack of rigorous assessments of content quality [11]. An evaluation of 10 pregnancy and childbirth-related apps in Australia found them to be highly useful in providing health information and education, monitoring various health-related behaviors, and offering advice, tips, and strategies. Furthermore, a 2-year follow-up evaluation of updates and content changes showed an increase in quality assessment scores, indicating an improvement in the quality of the apps [13].

Previous research examining the features of pregnancy and childbirth apps available in app stores, as well as their quality, has been conducted in the United States, Spain, and Australia [1,10-12]. The most frequently addressed topics were “weight gain,” “nutrition,” “fetal development,” “physical activity,” and “changes during pregnancy,” with the primary usage being self-monitoring or goal setting [1,11]. Past studies have shown that app usage can effectively enhance health behaviors such as improving knowledge, promoting physical activity, and encouraging a healthy diet among pregnant women. One study underscored the positive influence of apps on behavioral changes, citing an app designed to promote weight gain and increased consumption of fruits and vegetables during pregnancy, which had beneficial effects on childbirth outcomes [12]. The majority of previous studies evaluating apps were systematic reviews [1,11-13] of studies that implemented interventions using existing or newly developed apps, based on their intended purpose, and confirmed their effectiveness. Despite the growing number of health apps related to pregnancy and childbirth in South Korea’s mobile app market, most apps are used without any verification of their effectiveness [20].

In South Korea, research has been conducted on the quality assessment of apps designed for patients with hypertension or diabetes mellitus. However, it is challenging to find studies that explore the features of pregnancy/childbirth-related apps or evaluate their quality using standardized tools. Consequently, this study sought to identify pregnancy/childbirth-related apps through a systematic search in the South Korean app market, examine their characteristics, and evaluate their quality from both subjective and objective perspectives. This study aims to provide a foundation for selecting apps that offer accurate and appropriate information for expectant mothers.

The aim of this study was to explore the features of mobile apps pertaining to pregnancy or childbirth that are available in South Korea, and to evaluate their quality.

## Methods

**Ethics statement:** This study was exempted by the Institutional Review Board of Chungnam National University as it evaluated the quality of mobile apps currently in use.

### Study design

This descriptive study was conducted to assess the quality of apps related to pregnancy or childbirth.

### Study sample

The study sample consisted of pregnancy and childbirth-related apps found in the mobile app markets of iTunes and Google Play Store in South Korea. The apps selected for this study were those that were free, contained content related to pregnancy or childbirth and were available in the Korean language. In cases where an app was listed in both app stores under the same name, only one was chosen for the study. Apps were excluded from the study if they could not be downloaded due to technical issues, required payment, lacked pregnancy or childbirth-related content, were classified as games or entertainment without any educational or health-related purpose, were not relevant to pregnancy or childbirth, were solely designed to track menstruation and ovulation, or included in-app purchases such as games, shopping features, or ad-supported community apps.

From April 15 to April 29, 2022, the keywords “pregnancy” and “childbirth” were used to search the android app store (Google Play Store) and the iOS(iTunes). This search yielded names, categories, and descriptions, along with photos of various apps. In total, 201 apps were found in the android app store and 175 in the iOS store. Of these, 18 were paid apps, with 10 found in the android store and eight in the iOS store. Additionally, 52 apps were unrelated to the topic, with 49 in the android store and three in the iOS store. Furthermore, 16 apps were not available in Korean, with 14 in the android store and two in the iOS store. After applying selective criteria, a total of 290 apps (128 from the android store and 162 from the iOS store) were chosen for the initial analysis. There were 27 apps that appeared in both app stores. Given the prevalence of the android system in South Korea, these 27 duplicated apps were included in the android app store list, and their counterparts in the iOS were removed.

**Figure 1.**
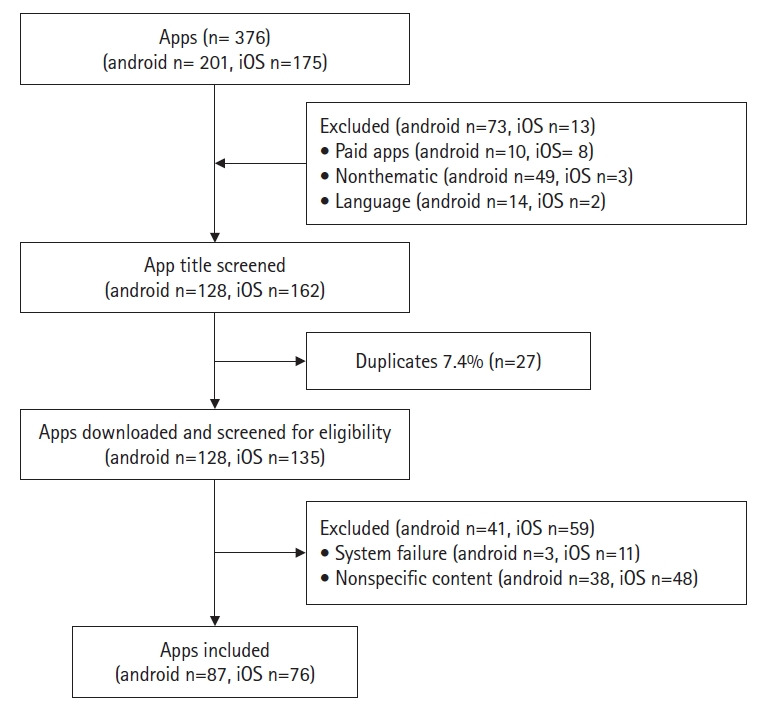
Flow sheet for the application (app) selection process.

In the second phase of analysis, 263 apps were selected for review: 128 from the android store and 135 from the iOS store. Each app was individually examined to determine if it met the exclusion criteria. Fourteen malfunctioning apps were excluded, three from the android store and 11 from the iOS store. Additionally, 86 apps with irrelevant content were also excluded: 38 from the android store and 48 from the iOS store. Consequently, the final analysis included a total of 163 apps: 87 from the android store and 76 from the iOS store (Figure 1).

### Instruments

#### Quality of mobile applications

The Mobile Application Rating Scale (MARS), developed by Stoyanov et al. [20] and subsequently translated into Korean, was utilized to measure the quality of mobile apps. The strength of MARS lies in its multidimensional approach to app assessment and the fact that it was designed based on a comprehensive review of the literature [21]. MARS is divided into two sections: a basic section, which evaluates the fundamental characteristics of apps (including app classification and quality ratings) and an app-specific section, which examines additional aspects related to the impact of apps on users’ health behaviors.

The section on app classification encompasses focus, theoretical background/strategies, age group, and technical aspects of the app. However, this study only scrutinized focus, which is composed of 12 items that an app targets (increase happiness/well-being, mindfulness/medication/relaxation, reduce negative emotions, depression, anxiety/stress, anger, behavior change, alcohol/substance use, goal setting, entertainment, relationships, physical health, and others).

The section on app quality ratings comprised a total of 23 items. These were divided into an objective assessment across four dimensions (engagement, functionality, aesthetics, and information) and a subjective assessment. The engagement dimension included five items: entertainment, interest, customization, interactivity, and target group. The functionality dimension was made up of four items: performance (accuracy and speed), ease of use, navigation, and gestural design. The aesthetics dimension, with three items, covered layout, graphics, and visual appeal. The information dimension, the largest with seven items, evaluated the accuracy of the app description, goals, quality and quantity of information, visual information, credibility, and evidence base. Each item was rated on a 5-point scale, ranging from inappropriate (1 point) to very good (5 points), or from strongly disagree (1 point) to strongly agree (5 points). The overall quality assessment score, which ranged from 4 to 20 points, was calculated by adding up the mean scores of the four dimensions (each ranging from 1 to 5 points). Higher scores in each dimension of the objective assessment section indicated better app quality. In this study, item number 19 in the information dimension (“Has the app been trialed/tested; must be verified by evidence in published scientific literature?”) was excluded from the score calculation. This was due to the lack of available information in the apps included in this study that could be used to assess this item. The subjective assessment included four items (recommendation, intention to continue use, intention to purchase, and overall assessment) to gauge satisfaction with the app. While the intention to purchase was scored at 1, 3, and 5 points, the other three items were scored on a 5-point scale (1 to 5 points). The mean of the four item scores (ranging from 1 to 5 points) was then calculated. A higher score in the subjective assessment indicated greater satisfaction with the app. MARS demonstrated high internal consistency (α=.90) and interrater reliability of r=.79 [21]. Following the precedent set by a previous study [20], which established a midpoint of 3 points on a 5-point scale (1 to 5 points) as a criterion, scores greater than 3.0 were assessed as desirable. This criterion was also applied in the current study. The app-specific section categorized the purpose of app use into six categories (awareness, knowledge, attitudes, intention to change, help seeking, and behavior change).

While the original creators of MARS did not stipulate the number of raters needed, it is crucial that if multiple raters are involved, they should have a comprehensive understanding of the MARS items and their relevance to the app themes [21]. In this study, we followed the procedure for ensuring interrater reliability [22]. Two researchers specializing in women’s health nursing used all the apps for a minimum of 2 weeks and independently evaluated them using the assessment tool. Subsequently, the assessment results from the two raters were compared. In cases where the assessment scores differed, a consensus was reached through the presentation of evidence and discussion of validity. This study ensured reliability with interrater reliability scores of r=.71 and r=.72 for the apps in the android and iOS stores, respectively.

#### Mobile application characteristics

The characteristics of the apps included the app stores selling them, the category suggested by the developer based on the content, the oversight of the authority, and the update frequency (less than 6 months, 6 months to less than 1 year, and 1 year or longer). Nine categories were discerned by examining the details registered by the developer in the app stores. However, the categories registered by the developer were not specifically categorized, and there were instances of duplication or ambiguity due to the developer’s arbitrary classification. As a result, the researchers of this study restructured and categorized them into “health/exercise,” “childbirth/parenting,” “role-playing/simulation,” “lifestyle/social networking,” “family,” and “information” based on the app content. During this recategorization, the “medical care” category was frequently identified in the app stores. However, the “childbirth/parenting” category was distinctly categorized in the android app store, providing android users with more specific pregnancy-related categories than the iOS. Conversely, the categorization was not as clear in the iOS, as apps related to pregnancy/childbirth were grouped under the “medical care” category. While the “childbirth/parenting” category included the “information” dimension, apps that contained records and behavioral changes related to childbirth and parenting were classified under the childbirth/parenting category. In contrast, the “information” category included apps that simply provided newsletters or support programs for pregnant women.

### Data collection and data analysis

The research team examined the features of the apps chosen for this study through a questionnaire. The quality of these apps was independently evaluated by two raters using a mobile app quality assessment tool. The scores from each rater were compared, and if a discrepancy of 3 points or more was found, the raters discussed their findings and reached a consensus on the final score. The data were analyzed using SPSS for Windows (ver. 26; IBM Corp., Armonk, NY, USA). A frequency analysis was performed on the app characteristic variables, and descriptive statistics were used for the total score and each dimension score of the app quality ratings. To compare quality assessment scores by category, additional tests such as the t-test, one-way analysis of variance, and *post hoc* test (when necessary) were conducted.

## Results

### Characteristics of apps related to pregnancy and childbirth

Out of 163 apps related to “pregnancy” and “childbirth,” 60 were exclusively registered on the android app store, while 76 were only available on the iOS. Additionally, 27 apps were registered on both platforms. For the purpose of this study, apps that were duplicated across both platforms were included in the android app store’s list. The most common category was health/exercise (n=92, 56.4%), followed by childbirth/parenting (n=31, 19.0%), role-playing/simulation (n=23, 14.1%), lifestyle/social networking (n=9, 5.5%), family (n=6, 3.6%), and information (n=2, 1.4%).

Only four apps (2.9%) had been reviewed by credible authorities. Of the 87 android apps, three stated that they had consulted with experts from the Korean Society of Ultrasound in Obstetrics and Gynecology and the Korean Pediatric Society. Among the 76 Apple apps, one claimed to have received advice from a yoga expert. As of July 31, 2022, the average update cycle was 367.6 days, with 66.8% of apps being updated within a year. More apps did not offer in-app purchases (n=92, 56.5%) compared to those that did (n=71, 43.5%) (Table 1).

### Quality assessment of pregnancy-related mobile apps

In the categorization of apps by 12 focus areas, three areas (anxiety/stress, anger, and alcohol/substance use) had no corresponding apps. Of the remaining nine focus areas, the most prevalent category was enhancing happiness/well-being, with 54 apps accounting for 33.1% of the total. This was followed by action/change (n=34, 20.8%), game (n=19, 11.6%), goal setting (n=19, 11.6%), relationships (n=14, 8.5%), physical health (n=13, 7.9%), mindfulness/meditation/relaxation (n=5, 3.0%), reducing negative emotions (n=3, 1.8%), and depression (n=2, 1.7%) (Table 2).

The mean score for the objective quality assessment was 3.74±0.45. The subdomain with the highest mean score was aesthetics, scoring 3.90±0.52. This was followed by information (3.81±0.53), functionality (3.79±0.75), and engagement (3.46±2.38). All these scores exceeded the benchmark score of 3.0 out of 5.0, indicating that both the overall objective quality and the quality of each dimension were satisfactory. The mean score for the subjective quality assessment was 3.35±0.62. The highest mean score was for the intention to purchase, which was 3.56±0.98. This was followed by the intention for continuous use (3.52±0.68) and recommendation (3.35±0.62).

Among the six categories of app-specific usage purposes, knowledge (3.66±0.67) received the highest score. This was followed by help seeking (3.37±0.71), intention to change (3.16±0.45), attitudes (3.14±0.42), behavior change (3.11±0.93), and awareness (2.91±0.59) (Table 2).

### Quality assessment of mobile apps by characteristics

The total score of objective quality assessment significantly differed according to the content category (F=3.86, *p*=.003). The dimensions of engagement (F=3.75, *p*=.003), functionality (F=4.38, *p*=.001), and aesthetics (F=2.63, *p*=.026) also showed significant differences. However, the post hoc test did not reveal any significant differences in group comparisons. When examining the quality assessment scores for the engagement dimension by app category, apps within the childbirth/parenting category (4.13±0.50) and family category (4.10±0.55) scored higher, while those in the information category (2.90±1.55) scored the lowest. In the functionality dimension, apps in the childbirth/parenting category (4.13±0.62) and role-playing/simulation category (4.10±0.48) scored higher, while those in the information category (2.87±0.88) scored the lowest. In the aesthetics dimension, apps in the childbirth/parenting category (4.04±0.45) scored the highest, while those in the information category (3.00±1.41) scored the lowest. There were no significant disparities in the subjective assessment scores when categorized based on app content. Apps within the childbirth/parenting category (14.55±1.67) and the family category (14.50±1.51) demonstrated higher overall subjective assessment scores. Conversely, apps in the information category (11.50±6.36) exhibited the lowest score.

There were no significant differences in the subjective assessment scores when categorized based on app content. Apps within the childbirth/parenting category (14.55±1.67) and the family category (14.50±1.51) demonstrated higher overall subjective assessment scores. Conversely, apps in the information category (11.50±6.36) exhibited the lowest score.

Additionally, there were no significant differences observed between the objective and subjective assessment scores regarding the supervision of an authority, up-to-date content, and in-app purchases (Table 3).

## Discussion

This study evaluated the objective and subjective quality of pregnancy and childbirth-related apps available in the android and iOS in South Korea. The results confirmed that the quality of these apps was desirable, exceeding the benchmark score of 3.0 proposed by a prior study [20].

The apps were updated on average every 367 days, with a significant variation in update frequency ranging from as little as 10 days to as much as 2,192 days. Apps related to community, music, and pregnancy diaries were updated within a 30-day period, while 33.2% of apps took more than a year to receive an update. This suggests that users should verify the timeliness of the information provided. Furthermore, only 2.9% of apps provided information about regulatory oversight, which could raise concerns about their credibility. As such, users should check for the presence of credibility indicators, and developers should include reviewer information to assure users of their app’s credibility.

The apps included in this study had an objective quality score of 3.86 points, suggesting a higher quality of pregnancy and childbirth-related apps in Korea compared to the 2.94 points scored in a similar Spanish study [11]. This discrepancy in quality assessment scores may be attributed to the nature of the app content. The higher score in this study could be due to the inclusion of apps that addressed both the physical and mental aspects of pregnancy and childbirth. In contrast, the previous study [11] focused on apps related to physical activities during pregnancy, reflecting the specificity of the content. In this study, the function dimension, one of the objective quality assessment dimensions, received the highest quality assessment score. This finding aligns with the Spanish study [11], where the function dimension also scored highest (4.00 points). However, this study demonstrated higher scores in the aesthetics, engagement, and information dimensions than those in the Spanish study (aesthetics, 3.00 points; engagement, 2.60 points; and information, 2.60 points). The high score in the function dimension in the Spanish study could be due to the selection of apps that promote changes in physical activity and positive lifestyle information during pregnancy, which can potentially enhance maternal and fetal health. Conversely, negative pregnancy outcomes can adversely affect maternal behavior changes. Meanwhile, the information dimension received the lowest scores in both this study and the Spanish study. This could be because the information dimension lacked sufficient items to evaluate whether the apps were well-sourced, used as academic resources, included reliable visuals, were regularly updated, or were reviewed by a reliable authority during development.

Significant differences were observed in the total objective quality assessment scores, as well as the scores for engagement, function, and aesthetics dimensions, based on the app content category. Specifically, apps in the childbirth/parenting, family, and role-playing/simulation categories, which garnered high user interest, scored highly in the objective quality assessment. The engagement dimension scores were higher for apps in the childbirth/parenting, family, role-playing/simulation, and lifestyle/social networking categories. This is likely to have been because apps in the childbirth/parenting and family categories offer features that allow mothers and partners to engage by monitoring their baby’s status and sharing information during pregnancy. Additionally, apps in the role-playing/simulation and lifestyle/social networking categories provide numerous opportunities for direct user participation. High scores in the aesthetics dimension were found among apps in the childbirth/parenting and role-playing/simulation categories, underscoring the importance of aesthetic appeal in these categories. Finally, the function dimension scores were higher for apps in the childbirth/parenting, family, and role-playing/simulation categories. This could be attributed to the fact that these apps offer both functionality and information. For instance, they may use three-dimensional technology to provide pregnancy-related information, animate the childbirth process in a flash format, or allow users to visually track their status by entering their gestational weeks or other information.

Nurses can recommend pregnancy and childbirth-related apps to pregnant women as educational tools. These apps can help track health status, provide basic information, and visually depict the fetus’s condition, location, and size. However, if the quality of these mobile apps is not assured, they could pose risks to the health management and healthy lifestyle of pregnant women. Therefore, nurses should guide pregnant women through the process of verifying the currency and expertise of apps via the app information before installation. This ensures the use of safe and reliable health management resources for both the pregnant women and their fetuses. To enhance the reliability of app information, it is crucial for app developers to collaborate with clinical experts. This collaboration can help organize useful content and ensure expert supervision. Developers should also aim to categorize apps clearly based on content and guarantee that the information is evidence-based and current. This will ensure that pregnancy and childbirth-related apps can be used effectively. Users who choose and utilize these apps should select those that provide up-to-date information by checking for regular updates. If they discover issues regarding the quality and quantity of information in the app content, they should raise their concerns with the app developers. Additionally, they should seek advice from healthcare providers involved in the pregnancy and childbirth process.

This study underscores the necessity of employing high-quality apps in practice to offer a range of informed medical services to pregnant women. Furthermore, it is crucial to carry out qualitative studies to verify the impact of app usage on the health management of pregnant women. Future research should also include quantitative studies on their experiences with app usage.

## Figures and Tables

**Table 1.** Characteristics of the pregnancy-related mobile applications (app) (N=163)

Characteristics	Categories	n (%)
Operating system	Android	87 (53.3)
	iOS	76 (46.7)
Categories	Health/exercise	92 (56.4)
	Childbirth/parenting	31 (19.0)
	Role-playing/simulation	23 (14.1)
	Lifestyle/Social networking	9 (5.5)
	Family	6 (3.6)
	Information	2 (1.4)
Reliable authority	Yes	4 (2.5)
	No	159 (97.5)
Update cycle	<6 months	82 (50.3)
	6 months to 1 year	9 (5.6)
	>1 year	72 (44.1)
In-app purchases	Yes	71 (43.5)
	No	92 (56.5)

**Table 2.** Quality evaluation of pregnancy-related mobile applications (apps) (N=163)

Characteristics	Categories	n (%) or mean±SD	Item mean±SD
Focus	Increasing happiness/well-being	54 (33.1)	
	Action/change	34 (20.8)	
	Game	19 (11.6)	
	Goal setting	19 (11.6)	
	Relationship	14 (8.5)	
	Physical health	13 (7.9)	
	Mindfulness/meditation/relaxation	5 (3.0)	
	Reducing negative emotions	3 (1.8)	
	Depression	2 (1.7)	
Objective quality	Engagement		3.46±2.38
	Functionality		3.79±0.75
	Aesthetics		3.90±0.52
	Information		3.81±0.53
	Total	3.74±0.45	
Subjective quality	Recommendation		3.35±0.62
	Continuous use		3.52±0.68
	Purchase		3.56±0.98
	Comprehensive evaluation		2.61±0.86
	Total	3.35±0.62	
App-specific goals	Knowledge		3.66±0.67
	Help seeking		3.37±0.71
	Intention to change		3.16±0.45
	Attitude		3.14±0.42
	Behavior change		3.11±0.93
	Awareness		2.91±0.59

**Table 3.** Differences in scores of objective and subjective quality by application (app) characteristics (N=163)

Characteristics		Objective quality										Subjective quality, total	
		Engagement		Functionality		Aesthetics		Information		Total			
		Mean±SD	F (*p*)	Mean±SD	F (*p*)	Mean±SD	F (*p*)	Mean±SD	F (*p*)	Mean±SD	F (*p*)	Mean±SD	F (*p*)
Content category	Health/exercise	3.58±0.75	3.75 (.003)	3.79±0.44	4.38 (.001)	3.73±0.44	2.63 (.026)	3.39±0.51	1.85 (.106)	63.76±8.85	3.86 (.003)	13.04±2.66	2.00 (.082)
	Childbirth/parenting	4.13±0.50		4.13±0.62		4.04±0.45		3.45±0.51		70.33±7.71		14.55±1.67	
	Role-playing/ simulation	3.98±0.61		4.10±0.48		3.86±0.66		3.44±0.71		68.55±9.24		13.15±2.23	
	Lifestyle/social networking	3.86±0.75		3.83±0.72		3.74±0.34		3.38±0.47		66.22±11.04		13.33±2.23	
	Family	4.10±0.55		3.87±0.51		3.94±0.74		3.42±0.35		69.16±8.97		14.50±1.51	
	Information	2.90±1.55		2.87±0.88		3.00±1.41		2.50±1.17		50.00±22.62		11.50±6.36	
Update cycle	<6 months	3.74±0.69	1.32 (.269)	3.81±0.52	3.01 (.053)	3.72±0.59	3.07 (.050)	3.23±0.56	1.29 (.278)	64.67±9.52	0.91 (.652)	13.44±2.63	0.02 (.972)
	6 months to 1 year	4.17±0.71		4.16±0.46		4.14±0.52		3.50±0.76		71.40±9.34		13.44±3.00	
	>1 year	3.79±0.84		3.98±0.48		3.85±0.36		3.36±0.61		66.69±9.50		13.33±2.39	
Reliable authority	Yes	4.05±0.85	0.70 (.480)	4.06±0.68	0.63 (.527)	4.00±0.72	0.71 (.476)	3.12±0.20	0.38 (.727)	65.93±9.39	0.06 (.802)	4.19±2.09	0.81 (.473)
	No	3.79±0.75		3.90±0.53		3.82±0.53		3.08±1.19		67.00±14.58		2.45±0.21	
Paid app	Yes	3.78±0.71	0.01 (.993)	3.91±0.49	0.23 (.813)	3.81±0.06	0.01 (.985)	3.33±0.47	0.52 (.599)	66.16±8.31	0.65 (.799)	13.30±0.28	0.37 (.516)
	No	3.78±0.77		3.89±0.54		3.81±0.56		3.28±0.66		65.84±10.26		13.59±0.33	
